# Domain IV of Annexin A5 Is Critical for Binding Calcium and Guarantees Its Maximum Binding to the Phosphatidylserine Membrane

**DOI:** 10.3390/molecules22122256

**Published:** 2017-12-19

**Authors:** Jie Wang, Jing Liu, Yulu Cao, Minjin Hu, Zichun Hua

**Affiliations:** 1 State Key Laboratory of Pharmaceutical Biotechnology, School of Life Sciences, Nanjing University, Nanjing 210023, China; 15850736984@126.com (J.W.); jingjingliua@163.com (J.L.); 2 Jiangsu TargetPharma Laboratories Inc., Changzhou High-Tech Research Institute of Nanjing University, Changzhou 213164, China; cyll2000@163.com (Y.C.); huminj98@163.com (M.H.); 3 Shenzhen Research Institute of Nanjing University, Shenzhen 518057, China

**Keywords:** annexin A5, domain IV, calcium, phosphatidylserine, binding

## Abstract

*Background*: Although domain IV of annexin A5 (anxA5) may be less effective in binding phosphatidylserine (PS), the four domains together may guarantee the maximum binding of anxA5 to the PS membrane. Additionally, previous research has shown that annexin mutants lacking one or more domain(s) have different biological activities compared to the wild-type. The present research mainly aims to study the role of domain IV in the crucial PS-binding function of anxA5. *Methods*: The domain IV-truncated anxA5 protein was constructed and purified. Isothermal titration calorimetry, flow cytometry and activated partial thromboplastin time were adopted to examine the function of domain IV in anxA5-PS binding directly or indirectly. *Results*: The domain IV-truncated form of anxA5 is impaired in binding PS liposome and apoptotic cells, and anticoagulation activity. The mutant cannot bind calcium, but binds PS only in the presence of calcium. *Conclusions*: Truncation of domain IV of anxA5 destroys its calcium-binding ability and impairs its PS-binding activity. Truncation of domain IV may induce conformation change of anxA5 or reduce the hydrophobic interactions between protein and membrane, which may explain the decrease of PS-binding affinity of the mutant.

## 1. Introduction

**Figure 1:**
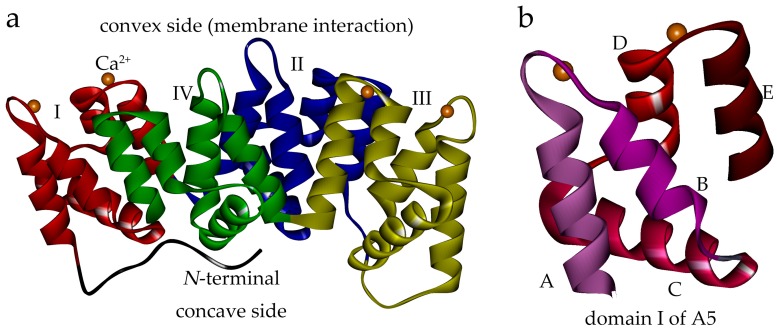
Human annexin A5 (anxA5) structure (PDB: 1ANX). (**a**) The anxA5 is structurally comprised of an N-terminal (black) and a C-terminal core composed of four domains. The convex side harbors calcium binding sites and is responsible for binding the membrane. The domains and Ca^2+^ are shown in different colors: domain I, red; domain II, blue; domain III, yellow; domain IV, green; Ca^2+^, orange balls. (**b**) Domain I of anxA5 as an example of the repeated domains. Each repeated domain is composed of five α-helices: A, B, C, D and E.

The annexins constitute a multi-member family characterized by the ability to bind acidic phospholipids in a calcium-dependent manner [1,2]. Each annexin protein usually comprises an N-terminal domain, and a C-terminal core domain composed of four (eight in annexin A6) 70-amino acid sub-domains [3,4]. The core forms a slightly bent disk shape, the convex side of which contains calcium and phospholipid binding sites, and is responsible for interaction with the membrane (Figure 1a). On the other hand, the N-terminal is mainly considered as the regulatory element in the structures and functions of annexin members [1,5].

Among all members, human annexin A5 (anxA5) deserves special attention, as it is widely applied in apoptosis detection both in vitro and in vivo, owing to its high affinity in binding phosphatidylserine (PS) exposed on apoptotic cells [6,7,8]. Additionally, anxA5 possesses various functions and applications [1,3,7,9,10,11,12,13,14], such as cell membrane repair, anticoagulation, in vivo therapy, and mediating targeted therapy. As a typical member of the annexins, anxA5 has the capacity for further development with respect to its potential clinical applications; particularly in the areas of in vivo apoptosis detection and therapy [7,12,15,16]. In general, the above functions and applications of anxA5 are closely related to its PS-binding ability, either directly or indirectly [1,3,7,9,10,11,12,13,14,17,18,19].

Previous studies have shown that domain I may play a prominent role in the PS-binding process, while domain III and IV have a limited effect [20,21,22]. Although domain III and IV are less effective in the direct binding between anxA5 and PS membrane, all four domains together guarantee the maximum binding of anxA5 to the membrane [20,21,23]. For example, Trp187 of domain III and Ser305 of domain IV stabilize the anxA5-membrane interaction by means of a hydrophobic ‘anchor’ and H-bonds, respectively [21]. The calcium binding site located in the loop between α-helices D/E of domain IV is also involved, with less efficiency, in inhibition of cPLA2 (cytosolic phospholipase A2) activity by depletion of phospholipids [22]. 

A mutant of annexin A2 (anxA2) lacking domain IV was observed to show a significant increase in binding of the CARDS (community-acquired respiratory distress syndrome) toxin compared to the wild-type [24]. Annexin A4 (anxA4) was found to possess a different alternatively spliced transcript, losing the majority of domain III and all of domain IV. This truncated anxA4 was translated and functionally analyzed, indicating that the protein binds to phospholipid vesicles in a calcium-independent manner, which is distinct from the wild-type. The existence of the novel form of anxA4 was considered to be of help in further defining the role of annexin members [25]. Additionally, other investigations concerning annexin mutants with one or more truncated domains have also been conducted, revealing the domain(s) responsible for specific biological functions or functional changes [26,27,28,29,30]. The existing results suggest that different domains may confer different biological activities to the members of annexins.

In this work, we aim to examine whether the deletion of domain IV will affect the functions, especially the central PS-binding function, of anxA5 through the in vitro activity assay of purified proteins. Together with previous research, this work may reveal the role of domain IV in the structure or function of anxA5 from a new viewpoint.

## 2. Results and Discussion

### 2.1. Successful Purification of AnxA5-Related Proteins 

**Figure 2:**
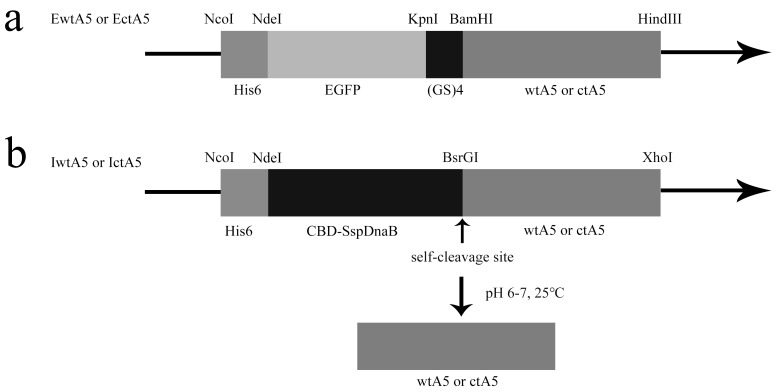
AnxA5 expression vectors. (**a**) EwtA5 and EctA5 protein expression vectors. The wtA5 or ctA5 coding sequence was fused to the C-terminal of EGFP via a (GS)_4_ linker, and the expressed proteins were able to be purified by Ni-affinity chromatography utilizing the His-tag. (**b**) IwtA5 and IctA5 protein expression vectors. The wtA5 or ctA5 coding sequence was fused to the C-terminal of CBD-SspDnaB. The expressed proteins were able to adsorb to the Ni-affinity resin, and the non-fusion protein wtA5 or ctA5 was obtained using the self-cleavage property of SspDnaB intein at its last residue. The restriction enzymes used, and the proteins or amino acid chains coded, are shown above and below the vectors, respectively. His_6_, His-tag for Ni-affinity chromatography; EGFP, enhanced green fluorescent protein; (GS)_4_, fusion protein linker; CBD-SspDnaB, intein.

Taking advantage of the His-tag in fusion proteins (Figure 2), EwtA5 (EGFP-fused wild-type anxA5), EctA5 (EGFP-fused C-terminal truncated anxA5), IwtA5 (Intein-fused wild-type anxA5) and IctA5 (Intein-fused C-terminal truncated anxA5) proteins adsorbed to the Ni-affinity resin. After washing with imidazole, the bound EwtA5 and EctA5 proteins were eluted using a high concentration of imidazole. The IwtA5 and IctA5 proteins were induced to undergo self-cleavage after buffer exchange (Figure 2b). The wtA5 and ctA5 proteins that were released flowed through and were collected. In detail, under the conditions of pH 6.0–7.0, 25 °C, the SspDnaB intein is able to catalyze self-cleavage at its last residue that was fused to the target protein. Under these cleavage conditions, the IwtA5 and IctA5 proteins split into two parts—intein and wtA5 (wild-type anxA5) or ctA5 (C-terminal truncated anxA5)—after self-cleavage (Figure 2b). After washing with HBS (10 mM HEPES, 140 mM NaCl, pH 7.4), the intein was still bound to the resin via the His-tag, while the wtA5 and ctA5 proteins flowed through. Through this mechanism, the non-fusion wtA5 and ctA5 proteins were indirectly obtained by affinity chromatography. 

**Figure 3:**
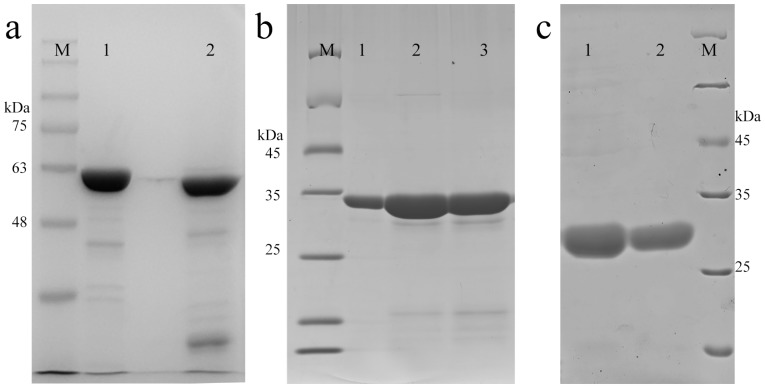
SDS-PAGE of purified proteins. (**a**) 10% SDS-PAGE of protein EwtA5 (lane 1) and EctA5 (lane 2). (**b**) 12% SDS-PAGE of protein wtA5. Lane 1: anxA5 standard; lane 2: flow through after cleavage of IwtA5; and lane 3: purified protein after buffer exchange. (**c**) 12% SDS-PAGE of protein ctA5. Lane 1: flow through after cleavage of IctA5; lane 2: purified protein after buffer exchange. M, protein marker.

As shown in Figure 3, the purified proteins were manifested on an SDS-PAGE gel, which was subsequently stained with Coomassie brilliant blue. The purities of EwtA5 and EctA5 were determined to be 90.9% and 85.5%, respectively (Figure 3a). The EwtA5 and EctA5 proteins obtained were 45 mg and 49 mg per liter of bacterial culture, respectively. The apparent relative MWs (molecular weight) of proteins were close to the theoretical MWs (EwtA5, 65.8 kDa; EctA5, 58.3 kDa). 

As shown in Figure 3b,c, wtA5 and ctA5 proteins were successfully purified with purities of 93.9% and 98.0%, respectively. The yield of wtA5 and ctA5 proteins was 12 mg and 6 mg per liter of bacterial culture, respectively. The apparent relative MWs of the proteins were consistent with the theoretical MWs (wtA5, 35.9 kDa; ctA5, 28.3 kDa) and related work [31,32]. This method for the production of non-fusion protein based on SspDnaB intein is convenient, and can produce proteins of high purity.

To conclude, the proteins were successfully purified, and could be applied for further investigation.

### 2.2. Truncation of Domain IV of AnxA5 Destroys Its Calcium-Binding Ability and Impairs Its Affinity for PS Liposome

Isothermal titration calorimetry (ITC) is a useful, label-free technique for characterizing the interaction mechanism of binding reactions from the perspective of thermodynamics. Since the interaction between anxA5 and PS is calcium-dependent, the binding between calcium and wtA5 or ctA5 was first measured using ITC.

**Figure 4:**
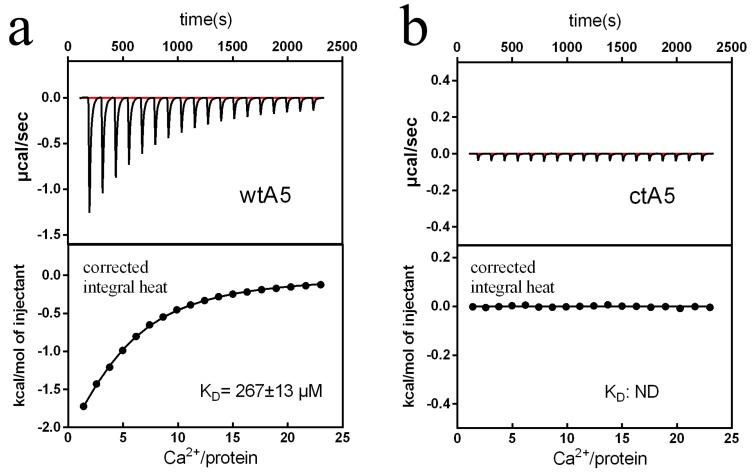
Titration of wtA5 and ctA5 with calcium. (**a**) Titration of 60 μM wtA5 with 7 mM calcium. (**b**) Titration of 60 μM ctA5 with 7 mM calcium. For each profile, the top shows the raw ITC data output during titration. The bottom represents the experimental points and the best fit. The points are derived from the raw data, and the heat of dilution was subtracted using the blank control of buffer titrated with calcium. The solid line at the bottom of each profile represents the best fit. *n* = 2.

During calcium titration, there was an obvious change in heat for wtA5, but not for ctA5 (Figure 4). The wtA5 protein binds to calcium with K_D_ = 267 μM, while there is no obvious binding reaction between ctA5 and calcium (Table 1), indicating that truncation of domain IV destroys the calcium-binding ability of anxA5. 

**Figure 5:**
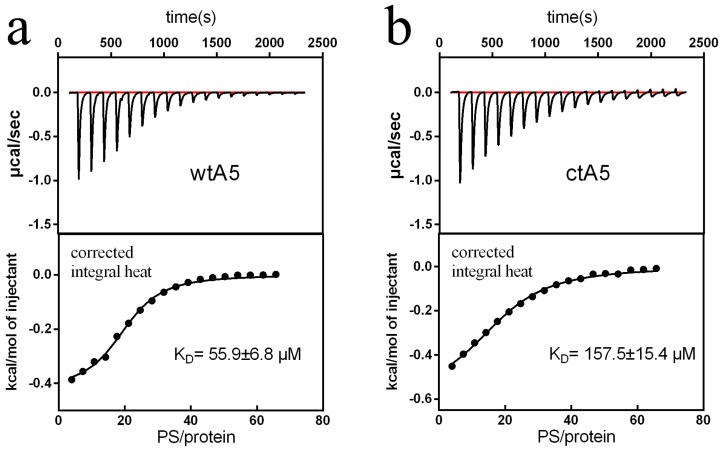
Titration of wtA5 and ctA5 with liposome in the presence of calcium. (**a**) Titration of 30 μM wtA5 with 25 mM POPS:POPC liposome. (**b**) Titration of 30 μM ctA5 with 25 mM POPS:POPC liposome. The top of each profile shows the raw ITC data output during titration. The bottom of each profile represents the experimental points and the best fit. The points are derived from the raw data, and the heat of dilution was subtracted using the blank control of buffer titrated with liposome. The solid line at the bottom of each profile represents the best fit.

**Figure 6:**
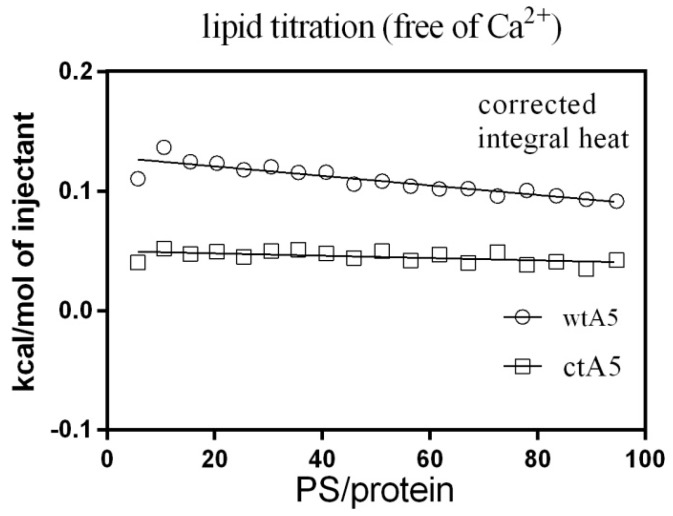
Titration of wtA5 and ctA5 with liposome in the absence of calcium. The data was processed and has been presented in the same way as indicated in Figure 5.

Subsequently, ITC was used to observe the binding between PS (POPS-containing liposome) and wtA5 or ctA5 protein, examining whether truncation of domain IV would affect the binding between them. There was an obvious change in heat during liposome titration in the presence of calcium (Figure 5), but not when calcium was absent (Figure 6), indicating that the interaction between liposome and wtA5 or ctA5 is calcium-dependent. The K_D_ of wtA5 or ctA5 binding to PS was 55.9 μM and 157.5 μM, respectively (Table 2), indicating that the affinity of ctA5 binding to PS is lower than wtA5. The ctA5 protein cannot bind calcium, but binds to PS-containing liposome only in the presence of calcium. These results indicate that the truncation of domain IV impairs the PS-binding affinity of anxA5. 

### 2.3. Truncation of Domain IV of AnxA5 Impairs Its Ability to Label Apoptotic Cells 

**Figure 7:**
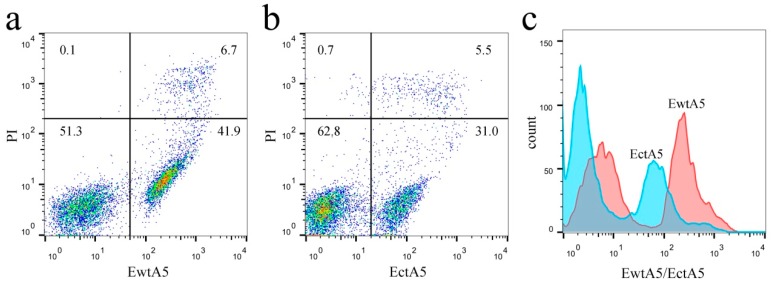
Apoptotic cells labeled with EwtA5 or EctA5 protein. (**a**) Dot plot of apoptosis-induced cells labeled with 25 nM EwtA5 and PI. The numbers shown in the quadrants represent the ratio of the cells. (**b**) Dot plot of apoptosis-induced cells labeled with 25 nM EctA5 and PI. The numbers shown in the quadrants represent the ratios of the cells, which are different from that of EwtA5 (*p* ≤ 0.01, *n* = 3). (**c**) Comparison between histograms of cells labeled with EwtA5 and EctA5. Compared to the histogram of cells labeled with EwtA5, there is an obvious movement to the left in that for EctA5.

AnxA5 proteins are widely applied in the field of apoptosis detection, utilizing its high affinity for PS expressed on apoptotic cells. To investigate the role of domain IV, a flow cytometry (FCM)-based apoptosis detection assay was used to estimate and compare the ability of EwtA5 and EctA5 proteins to label apoptotic cells. The EctA5-labeled cells showed a different distribution ratio compared with that of EwtA5 (Figure 7a,b). Statistical analysis (*p* ≤ 0.01, *n* = 3) revealed significant differences between the ratios. However, there was no significant difference (*p* = 0.1268) between the ratios of PI (propidium iodide)-positive cells. Thus, the differences in the distribution ratios of cells in Figure 7a,b were caused by the proteins but not PI. There is an obvious movement to the left in the histogram of cells labeled with EctA5 compared to that of EwtA5 (Figure 7c), indicating that the ability of EctA5 to label apoptotic cells has been impaired. 

The above FCM analysis indicates that truncation of domain IV impairs the ability of anxA5 to label apoptotic cells. 

### 2.4. Truncation of Domain IV Impairs the Anticoagulation Activity of AnxA5

**Figure 8:**
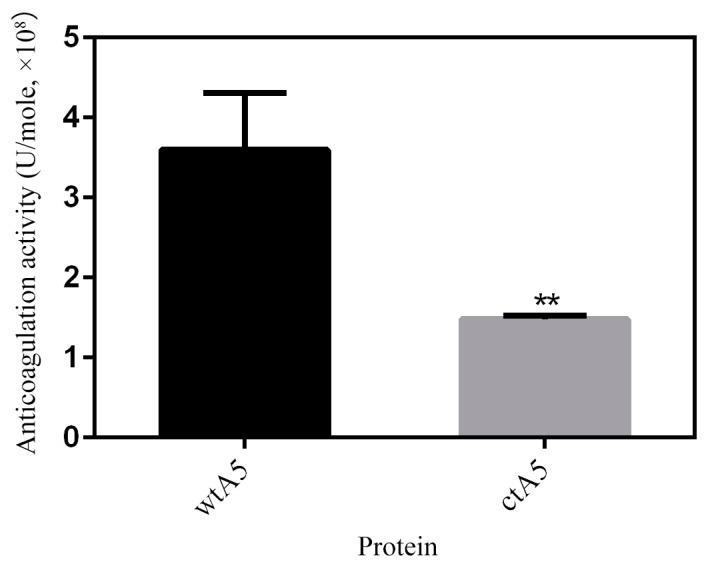
APTT assay of wtA5 and ctA5 proteins. The anticoagulation activity of ctA5 was significantly (*p* = 0.0068, *n* = 3) lower than that of wtA5.

AnxA5 protein has potent anticoagulation activity resulting from its PS-binding ability, blocking the availability of PS for coagulation reactions [33]. An APTT (activated partial thromboplastin time) assay was carried out to estimate the anticoagulation effect of proteins in the intrinsic pathway of coagulation. The anticoagulation activity of wtA5 and ctA5 was determined to be (3.60 ± 0.71) × 10^8^ and (1.48 ± 0.04) × 10^8^ U/mole respectively (Figure 8). The anticoagulation activity of ctA5 was significantly (*p* = 0.0068, *n* = 3) lower than that of wtA5, indicating that domain IV is important for the anticoagulation activity of anxA5.

The experiments above reveal the role of domain IV of anxA5 from different perspectives, including the binding of PS liposome and apoptotic cells, and its anticoagulation activity. The apoptosis detection application and anticoagulation activity of anxA5 are basically reflections of its PS-binding activity. Interestingly, ITC analysis showed that the domain IV-truncated form of the anxA5 protein does not bind calcium, but binds to PS liposome only in the presence of calcium. Together with the ITC experiments, the FCM analysis and APTT assay illustrate that, in nature, domain IV of anxA5 is essential for achieving its maximum PS binding. Additionally, domain IV is critical for anxA5 binding of calcium. 

Previous study has shown that a natural variant of anxA4, losing the majority of domain III and all of domain IV, binds to PS liposome in a calcium-independent way, which is distinct from the full length of anxA4 [25]. Functional analysis of several other mutants of anxA4 that lacked one or more domains indicates that domain III is required for its calcium-dependent binding activity for surfactant protein A [26]. Similar studies showed that the mutant of anxA2 that lacked domain IV had a higher affinity for CARDS toxin compared to the wild-type, which could possibly be caused by conformational changes [24]. Additionally, deletion of the last six domains of annexin A6 (anxA6) impairs related biological functions, such as coated pit budding activity and low-density lipoprotein degradation [27,28]. Truncation of the last five C-terminal domains of anxA6 was found to inhibit membrane repair in a manner different from the wild-type [30,34]. What’s more, the down-regulation of the short form of annexin A10 (anxA10), losing domain I and most of domain II, was associated with hepatocellular carcinoma [29,35]. These studies suggest that different domains may confer different biological activities to the members of the annexin group.

Individual domains and integral proteins may exhibit a broad range of diversity and specificity in both structure and function [36], which may also explain the variations in activity of annexin mutants lacking one or more domains. Even though the four domains in the core of annexins share an almost identical fold, they may exhibit different physicochemical properties when expressed as separate domains, such as domain IV being by far the most hydrophobic [37]. The single domain IV mutant (anxA2-Div mutant) of anxA2 was shown to compete with endogenous anxA2, interfering with VEGF-dependent network formation and disrupting the preformed capillary-like networks [38]. Additionally, the anxA2-Div mutant was able to bind to cognate mRNA of anxA2 [39]. 

Considering the research above, the reasons why truncation of domain IV led to the decrease of anxA5-PS binding affinity may be explained in terms of two aspects. Firstly, the anxA5 mutant lacking domain IV may undergo conformation change, leading to a loss of calcium-binding ability and a decrease in PS-binding affinity. Secondly, domain IV may contribute to anxA5-PS binding via hydrophobic interaction with the membrane, which could explain why there is an obvious decrease in entropy change (TΔS) during the ctA5-PS interaction.

Subsequent research could be conducted to study similar mutants lacking one or more domains, as well as investigating the domains individually, to further reveal the four domains from the perspective of their uniqueness, not just being repeats of annexin. 

## 3. Materials and Methods

### 3.1. Construction of EGFP-AnxA5 Fusion Protein Expression Vectors

The EGFP fragments were amplified with primers e-F and e-R1, the product of which was again amplified using primer pairs e-F and e-R2 (Table 3). The recovered fragments were inserted into pET28a (+) to construct pET28a (+)-His_6_-EGFP-(GS)_4_ (pHEGS). The fragments of wild-type anxA5 (wtA5) and its mutant (ctA5) lacking domain IV (the last 66 residues) were amplified using the primers indicated in Table 3. The fragments were inserted into pHEGS vectors to construct EGFP-wtA5 (EwtA5) and EGFP-ctA5 (EctA5) expression vectors. The EGFP-fused proteins were designed for flow cytometry analysis.

### 3.2. Construction of Intein-AnxA5 Fusion Protein Expression Vectors

The CBD-SspDnaB intein (pTWIN1 plasmid as template; Cat. No. N6951S, NEB, Beverly, MA, USA), wtA5 and ctA5 fragments were amplified using the primers indicated in Table 4. The intein fragments were inserted into pET28a (+) to construct vector pET28a (+)-His_6_-CBD-Intein (pHI). Subsequently, the wtA5 and ctA5 fragments were inserted into pHI to construct intein-wtA5 (IwtA5) and intein-ctA5 (IctA5) expression vectors. The intein fused vectors were designed to obtain non-fusion proteins wtA5 and ctA5 after self-cleavage of intein, as described below. The non-fusion proteins were designed for isothermal titration calorimetry analysis and anticoagulation assay.

The recognition sites of restriction enzymes used are shown in bold and underlined. All primers listed were ordered from GenScript (Nanjing, China), and plasmids were sent to GenScript for sequencing.

### 3.3. Expression and Purification of AnxA5 and Related Proteins

The above expression vectors were transformed into *E. coli* BL21 (DE3) and induced to express protein as previously reported [40]. The EGFP-fused anxA5 proteins were purified using Ni-affinity chromatography, while the intein-fused anxA5 proteins were purified using the same method with some modifications [40]. Briefly, the IwtA5 and IctA5 bacteria were suspended in lysis buffer (50 mM Tris-HCl, 500 mM NaCl, pH 8.5). After sonication and centrifugation, the supernatant was loaded onto a Ni-IDA affinity column and the column was washed with 50 mM imidazole in lysis buffer. To obtain non-fusion proteins wtA5 and ctA5, the column was washed with cleavage buffer (50 mM Tris-HCl, 500 mM NaCl, pH 6.0–7.0), after which the column was kept at 25 °C for 24 h. The protein was collected by washing the column with HBS (10 mM HEPES, 140 mM NaCl, pH 7.4). All the proteins purified were applied onto a polyacrylamide desalting column (Cat. No. 89893, Thermo Scientific, Shanghai, China) equilibrated with HBS. The collected fractions were stored at −80 °C until further analysis. 

### 3.4. SDS-PAGE Analysis

After quantification by bicinchoninic acid (BCA) staining, the purities of the proteins were analyzed by sodium dodecyl sulfate (SDS) polyacrylamide gel electrophoresis (PAGE) and stained with Coomassie brilliant blue.

### 3.5. Isothermal Titration Calorimetry Analysis

The isothermal titration calorimetry (ITC) analyses of non-fusion wtA5 and ctA5 proteins were performed on a MicroCal iTC200 (Malvern, UK). For calcium titration, calcium chloride solution (7 mM CaCl_2_ in HBS) was injected to the protein solution (60 μM protein in HBS) at 15 °C [41]. For lipid titration, the liposome composed of POPC:POPS (60:40 molar ratio; Avanti polar lipids, Alabaster, AL, USA) was prepared using sonication with HBS [42]. The protein solution (30 μM) filled in the sample cell was titrated with 25 mM liposome at 15 °C. In lipid titration experiments, both the protein and liposome solution were adjusted to have the same calcium concentration of 0.75 mM [41]. After titration, the data was analyzed using MicroCal ITC-ORIGIN analysis software using the one-site binding model.

### 3.6. Apoptosis Detection by Flow Cytometry

Jurkat cells were induced to undergo apoptosis by addition of etoposide (25 μM), and apoptosis detection was carried out as previously described [40]. The apoptosis-induced cells were incubated with 25 nM EwtA5 or EctA5 proteins for 30 min on ice. After adding 10 μL propidium iodide (PI; 50 μg/mL, Cat. No. 556463, BD Pharmingen, Shanghai, China) to the suspension, the cells were analyzed by FACSCalibur system (BD Biosciences, San Jose, CA, USA) immediately. The data was analyzed and processed as described [40].

### 3.7. Anticoagulation Assay

Activated partial thromboplastin time (APTT) was determined using a Thrombotimer (Behnk Elektronik, Germany) with an assay kit from Sunbio (Cat. No. Y4012, Shanghai, China) according to the manufacturer’s instructions. The goat plasma (50 μL) was mixed with purified non-fusion proteins or buffer (50 μL) and APTT reagent (50 μL) in a coagulation cup, followed by incubation for 5 min at 37 °C. Then, CaCl_2_ (0.025 M, 50 μL, preheated to 37 °C) was added to the cup, after which the clotting time was recorded. APTT results are expressed as U = (sample clotting time-buffer clotting time)/(buffer clotting time × protein molar number).

## 4. Conclusions

Based on previous investigations on anxA5 and other annexins, we successfully designed and purified domain IV-truncated anxA5 proteins. Through ITC, FCM, and APTT assays, we estimated the role of domain IV in anxA5-PS binding, either directly or indirectly. The results showed that domain IV is critical for anxA5 in binding calcium, and maximizes the binding between anxA5 and the PS membrane. Considering previous reports, domain IV may contribute to the interaction between anxA5 and PS through the influence of protein conformation, or a hydrophobic interaction between them. This work characterizes the role of domain IV in anxA5-PS binding, which is expected to help further define the relationship between the structures and functions of annexin members. 

## Figures and Tables

**Table 1:** Parametric analysis of calcium titration.

Ca^2+^ Titration	wtA5	ctA5
*N*	4.1 ± 0.3	ND
K (M^−1^)	3750 ± 188	ND
K_D_ (μM)	267 ± 13	ND
ΔH (kcal/mol)	−4.03 ± 0.28	ND
TΔS (kcal/mol)	0.68	ND
ΔG (kcal/mol)	−4.71 ± 0.28	ND

**Table 2:** Parametric analysis of liposome titration.

Liposome Titration	wtA5	ctA5
*N*	19.6 ± 0.4	18.5 ± 0.6
K (M^−1^)	17,900 ± 2160	6350 ± 614
K_D_ (μM)	55.9 ± 6.8	157.5 ± 15.4
ΔH (kcal/mol)	−0.42 ± 0.01	−0.60 ± 0.02
TΔS (kcal/mol)	4.67	3.89
ΔG (kcal/mol)	−5.09 ± 0.01	−4.49 ± 0.02

**Table 3:** Primers for EGFP fusion proteins.

Primer	5′ → 3′	Target
e-F	GGAATTC **CATATG** GTGAGCAAGGGCG	EGFP
e-R1	TTCCAGAACCGGTACCCTTGTACAGCTCGTCCAT	
e-R2	CGC **GGATCC** TGAGCCACTTCCAGAACCGGTAC	
ea5-F	CGC **GGATCC** ATGGCACAGGTTCTCAGAGG	forward, wtA5 and ctA5
ea5-wtR	CGTC **AAGCTT** ATTAGTCATCTTCTCCACAGAGC	reverse, wtA5
ea5-ctR	CGTC **AAGCTT** ATTAGGTCTCTGCAAGGTAGGCAG	reverse, ctA5

**Table 4:** Primers for intein fusion protein.

Primers	5′ → 3′	Target
i-F	GGAATTC **CATATG** AAAATCGAAGAAGGT	CBD-SspDnaB
i-R	CCG **CTCGAG** CCCATGGCTCTTCCGTTG	
ia5-F	ATCAT **TGTACA** CAACGCACAGGTTCTCAGAGGC	forward, wtA5 and ctA5
ia5-wtR	CCG **CTCGAG** TTATTAGTCATCTTCTCCAC	reverse, wtA5
ia5-ctR	CCG **CTCGAG** TTATTAGGTCTCTGCAAGGTAGGC	reverse, ctA5
